# The role of medical schools in UK students’ career intentions: findings from the AIMS study

**DOI:** 10.1186/s12909-024-05366-6

**Published:** 2024-05-31

**Authors:** Tomas Ferreira, Alexander M. Collins, Arthur Handscomb, Dania Al-Hashimi, the AIMS Collaborative, Tomas Ferreira, Alexander M. Collins, Rita Horvath, Oliver Feng, Richard J. Samworth, Mario K. Teo, Crispin C. Wigfield, Maeve K. Mulchrone, Alisha Pervaiz, Heather A. Lewis, Anson Wong, Buzz Gilks, Charlotte Casteleyn, Sara Kidher, Erin Fitzsimons-West, Tanzil Rujeedawa, Meghna Sreekumar, Eliza Wade, Juel Choppy-Madeleine, Yasemin Durmus, Olivia King, Yu Ning Ooi, Malvi Shah, Tan Jit Yih, Samantha Burley, Basma R. Khan, Emma Slack, Rishik S. Pilla, Jenny Yang, Vaishvi Dalal, Brennan L. Gibson, Emma Westwood, Brandon S. H. Low, Sara R. Sabur, Wentin Chen, Maryam A. Malik, Safa Razzaq, Amardeep Sidki, Giulia Cianci, Felicity Greenfield, Sajad Hussain, Alexandra Thomas, Annie Harrison, Hugo Bernie, Luke Dcaccia, Linnuel J. Pregil, Olivia Rowe, Ananya Jain, Gregory K. Anyaegbunam, Syed Z. Jafri, Sudhanvita Arun, Alfaiya Hashmi, Ankith Pandian, Joseph R. Nicholson, Hannah Layton-Joyce, Kouther Mohsin, Matilda Gardener, Eunice C. Y. Kwan, Emily R. Finbow, Sakshi Roy, Zoe M. Constantinou, Mackenzie Garlick, Clare L. Carney, Samantha Gold, Bilal Qureshi, Daniel Magee, Grace Annetts, Khyatee Shah, Kholood T. Munir, Timothy Neill, Gurpreet K. Atwal, Anesu Kusosa, Anthony Vijayanathan, Mia Mäntylä, Momina Iqbal, Sara Raja, Tushar Rakhecha, Muhammad H. Shah, Pranjil Pokharel, Ashna Anil, Kate Stenning, Katie Appleton, Keerthana Uthayakumar, Rajan Panacer, Yasmin Owadally, Dilaxiha Rajendran, Harsh S. Modalavalasa, Marta M. Komosa, Morea Turjaka, Sruthi Saravanan, Amelia Dickson, Jack M. Read, Georgina Cooper, Wing Chi Do, Chiamaka Anthony-Okeke, Daria M. Bageac, David C. W. Loh, Rida Khan, Ruth Omenyo, Aidan Baker, Imogen Milner, Kavyesh Vivek, Manon Everard, Wajiha Rahman, Denis Chen, Michael E. Bryan, Shama Maliha, Vera Onongaya, Amber Dhoot, Catherine L. Otoibhi, Harry Donkin-Everton, Mia K. Whelan, Claudia S. F. Hobson, Anthony Haynes, Joshua Bayes-Green, Mariam S. Malik, Subanki Srisakthivel, Sophie Kidd, Alan Saji, Govind Dhillon, Muhammed Asif, Riya Patel, Jessica L. Marshall, Nain T. Raja, Tawfique Rizwan, Aleksandra Dunin-Borkowska, James Brawn, Karthig Thillaivasan, Zainah Sindhoo, Ayeza Akhtar, Emma Hitchcock, Kelly Fletcher, Lok Pong Cheng, Medha Pillaai, Sakshi Garg, Wajahat Khan, Ben Sweeney, Ria Bhatt, Madison Slight, Adan M. I. Chew, Cameron Thurlow, Kriti Yadav, Niranjan Rajesh, Nathan-Dhruv Mistry, Alyssa Weissman, Juan F. E. Jaramillo, William Thompson, Gregor W. Abercromby, Emily Gaskin, Chloe Milton, Matthew Kokkat, Momina Hussain, Nana A. Ohene-Darkoh, Syeda T. Islam, Anushruti Yadav, Eve Richings, Samuel Foxcroft, Sukhdev Singh, Vivek Sivadev, Guilherme Movio, Ellena Leigh, Harriet Charlton, James A. Cairn, Julia Shaaban, Leah Njenje, Mark J. Bishop, Humairaa Ismail, Sarah L. Henderson, Daniel C. Chalk, Daniel J. Mckenna, Fizah Hasan, Kanishka Saxena, Iona E. Gibson, Saad Dosani

**Affiliations:** 1 School of Clinical Medicine, University of Cambridge, Cambridge, UK; 2 School of Public Health, Faculty of Medicine, Imperial College London, London, UK; 3 Bristol Medical School, University of Bristol, Bristol, UK; 4 Sheffield Medical School, University of Sheffield, Sheffield, UK

**Keywords:** NHS, Medical students, Career intentions, AIMS study, Medical school, Health policy

## Abstract

**Objectives:**

To investigate differences in students’ career intentions between UK medical schools.

**Design:**

Cross-sectional, mixed-methods online survey.

**Setting:**

The primary study included all 44 UK medical schools, with this analysis comprising 42 medical schools.

**Participants:**

Ten thousand four hundred eighty-six UK medical students.

**Main outcome measures:**

Career intentions of medical students, focusing on differences between medical schools. Secondary outcomes included variation in medical students’ satisfaction with a prospective career in the NHS, by medical school.

**Results:**

2.89% of students intended to leave medicine altogether, with Cambridge Medical School having the highest proportion of such respondents. 32.35% of respondents planned to emigrate for practice, with Ulster medical students being the most likely. Of those intending to emigrate, the University of Central Lancashire saw the highest proportion stating no intentions to return. Cardiff Medical School had the greatest percentage of students intending to assume non-training clinical posts after completing FY2. 35.23% of participating medical students intended to leave the NHS within 2 years of graduating, with Brighton and Sussex holding the highest proportion of these respondents. Only 17.26% were satisfied with the prospect of working in the NHS, with considerable variation nationally; Barts and the London medical students had the highest rates of dissatisfaction.

**Conclusions:**

This study reveals variability in students’ career sentiment across UK medical schools, emphasising the need for attention to factors influencing these trends. A concerning proportion of students intend to exit the NHS within 2 years of graduating, with substantial variation between institutions. Students’ intentions may be shaped by various factors, including curriculum focus and recruitment practices. It is imperative to re-evaluate these aspects within medical schools, whilst considering the wider national context, to improve student perceptions towards an NHS career. Future research should target underlying causes for these disparities to facilitate improvements to career satisfaction and retention.

**Supplementary Information:**

The online version contains supplementary material available at 10.1186/s12909-024-05366-6.

## Introduction

The rapidly changing dynamics of modern healthcare require a comprehensive understanding of the driving forces behind the career trajectories of doctors. As the landscape of patient care, healthcare policy, and medical technology continues to evolve, so too do the career choices of emerging doctors. These choices, as research increasingly demonstrates, are not solely the product of personal inclination or market demand but are deeply influenced by their experiences in medical school [1].

In recent years, the recruitment and retention of doctors within the United Kingdom’s (UK) National Health Service (NHS) have emerged as pressing concerns, requiring a detailed analysis of the factors influencing the career intentions of medical students [2–4]. To address this, the Ascertaining the career Intentions of Medical Students (AIMS) study — the largest ever UK medical student survey — delineated the career intentions and underlying motivations of students, highlighting a significant trend towards alternative careers or emigration, influenced predominantly by remuneration, work-life balance, and working conditions within the NHS [5].

Expanding upon the insights of the AIMS study, we seek to further explore the nuanced differences in career intentions among medical students, in relation to their institutional affiliations, and foster a dialogue concerning medical education and workforce planning in the UK, highlighting the role of medical schools in shaping career trajectories. It is posited that these educational institutions, with their diverse curricular designs and teaching philosophies, may play a pivotal role in shaping the prospective professional trajectories of their students. Furthermore, the distinct socio-economic and cultural environments in which these schools are situated, and those of the students they attract, may also contribute to the varied perspectives and career aspirations of students. Historically, the field of medical education has been subject to a variety of pedagogical philosophies, curricular reforms, and institutional priorities. These variations across medical schools, while often subtle, can result in significant differences in the way students perceive their roles, responsibilities, and opportunities within the broader healthcare ecosystem. Literature suggests that various elements including the culture of a medical school and its sociocultural context play a significant role in shaping the professional aspirations of its students [1, 6].

This manuscript seeks to identify and characterise these differences, with a focused analysis on how various medical schools in the UK might be influencing the career preferences and intended paths of their students. These findings may hold significant implications for various stakeholders within the healthcare sector. Policymakers could find guidance for strategic investments and resource allocation to areas anticipated to experience shortages, while educationalists could gain an opportunity for reflection on the potential influence of their institutions on student aspirations, thereby considering necessary adjustments. Furthermore, it affords insights for improved recruitment strategies, critical to ensuring the NHS’s continued role in the UK.

## Methods

### Study design

The AIMS study was a national, cross-sectional, multi-centre study of medical students conducted according to its published protocol and extensively described in its main publication [5, 7]. Participants from 44 UK medical schools recognised by the General Medical Council (GMC) were recruited through a non-random sampling method via a novel, self-administered, 71-item questionnaire. The survey was hosted on the Qualtrics survey platform (Provo, Utah, USA), a GDPR-compliant online platform that supports both mobile and desktop devices.

### Participant recruitment and eligibility

In an attempt to minimise bias and increase the survey’s reach to promote representativeness, a network of approximately 200 collaborators was recruited across 42 medical schools – one collaborator per year group, per school – prior to the study launch to disseminate the study. All students were eligible to apply to become a collaborator. This approach aimed to obtain a representative sample and improve our findings’ generalisability. The survey was disseminated between 16 January 2023 and 27 March 2023, by the AIMS Collaborative via social media (including Instagram, Facebook, WhatsApp, and LinkedIn), word of mouth, medical student newsletters/bulletins, and medical school emailing lists.

Individuals were eligible to participate in the survey if they were actively enrolled in a UK medical school acknowledged by the GMC and listed by the Medical School Council (MSC). Certain new medical schools had received approval from the GMC but were yet to admit their inaugural cohort of students, so were excluded from the study.

### Data processing and storage

To prevent data duplication, each response was restricted to a single institutional email address. Any replicated email entries were removed prior to data analysis. In cases where identical entries contained distinct responses, the most recent entry was kept. Responses for which valid institutional email addresses were missing were removed prior to data analysis to preserve the study’s integrity.

The findings of this subanalysis, and the AIMS study, were reported in accordance with the STROBE (Strengthening the Reporting of Observational Studies in Epidemiology) guidelines [8].

### Quantitative data analysis

Descriptive analysis was carried out with Microsoft Excel (V.16.71) (Arlington, Virginia, USA), and statistical inference was performed using RStudio (V.4.2.1) (Boston, Massachusetts, USA). Tables and graphs were generated using GraphPad Prism (V.9.5.0) (San Diego, California, USA). ORs, CIs and *p* values were computed by fitting single-variable logistic regression models to explore the effect of various demographic characteristics on students’ career intentions. CIs were calculated at 95% level. We used *p* < 0.05 to determine the statistical significance for all tests.

### Study population and exclusion

All current students of all year groups at UK medical schools recognised by the GMC and the MSC were eligible for participation. Brunel Medical School and Kent and Medway Medical School were excluded from this current analysis due to the limited number of respondents from these institutions (*n* < 30), to avoid misrepresenting the career intentions and characteristics of their broader student populations.

### Ethical approval

Ethical approval was granted by the University of Cambridge Research Ethics Committee (reference PRE.2022.124) on the 5th of January 2023. Prior to completing the survey, all participants provided informed consent. Participating medical schools were contacted prior to data collection to seek support and request permission to contact their students.

## Results

### Demographics

In total, 10,486 students across all 44 UK medical schools participated in the survey. To enable comparison of students’ career intentions between medical schools, only 42 medical schools were considered due to the sample size gathered. The average number of responses per medical school was 244, with a median of 203 (IQR 135–281). Participants had a median age of 22 (IQR 20–23). Among the participants, 66.5% were female (*n* = 6977), 32.7% were male (*n* = 3429), 0.6% were non-binary (*n* = 64), and 16 individuals chose not to disclose their gender. A detailed breakdown of participant characteristics, including gender, ethnicity, previous schooling, and course type, is illustrated in Supplemental Figs. 1a-d.

**Fig. 1:**
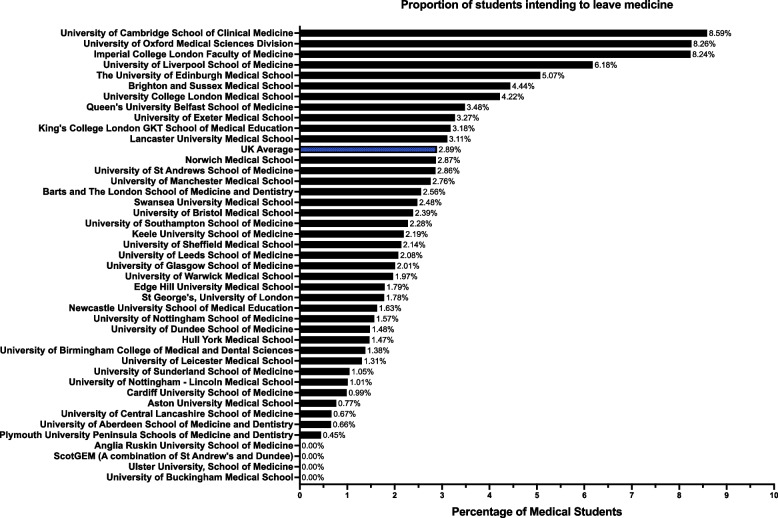
Proportion of Medical Students Intending to Leave the Profession Across UK Medical Schools. The figure depicts the percentage of students at each UK medical school who intend to exit the medical field entirely. Percentages are calculated as a proportion of total respondents from each individual school

A total of 303/10,486 (2.89%, CI: 2.59, 3.23%) medical students intended to leave the profession entirely, either immediately after graduation (*n* = 104/303, 34.32%, CI: 29.20, 39.84%), after completion of FY1 (*n* = 132/303, 43.56%, CI: 38.1, 49.19%), or after completion of FY2 (*n* = 67/303, 22.11%, CI: 17.8, 27.12%). Figure 1 illustrates the distribution of these students throughout UK medical schools as a percentage of total response numbers per school. The medical schools of Cambridge, Oxford, and Imperial College medical schools had the highest proportion of students intending to leave the profession altogether.

**Fig. 2:**
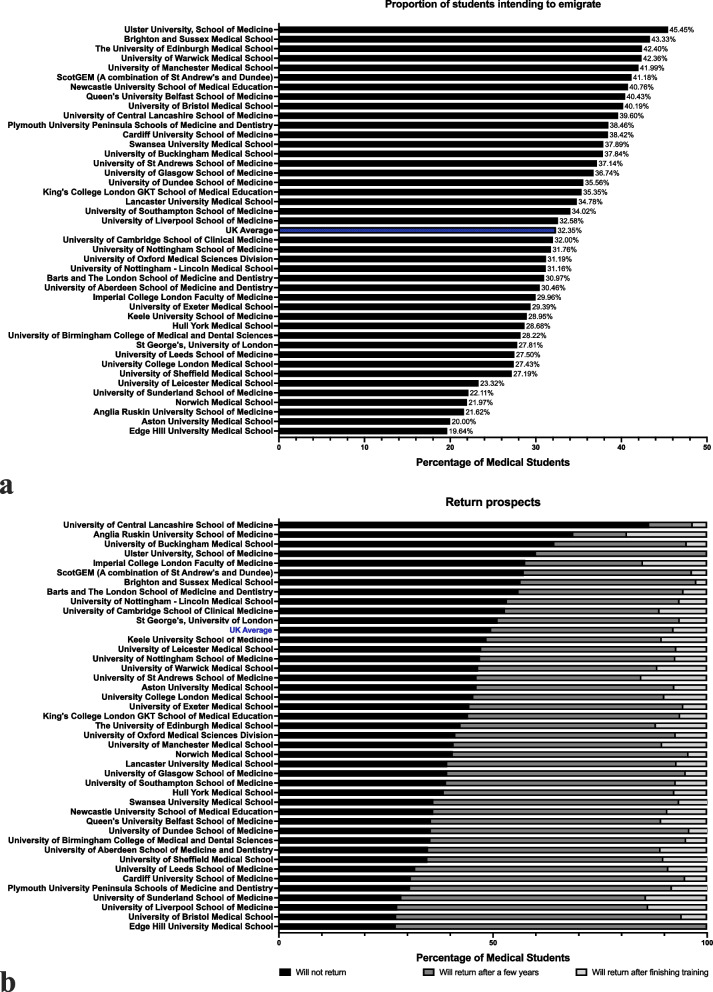
Proportion of Medical Students Intending to Emigrate Across UK Medical Schools (a) and Return Prospects (b). **a** illustrates the proportion of students from each UK medical school who intend to emigrate for medical practice, relative to total respondents from each school. **b** delineates the return prospects among students planning to emigrate

Furthermore, 32.35% of participating medical students (*n* = 3392/10,486, CI: 31.46, 33.25%) expressed intentions to emigrate to practise medicine, either immediately after graduation (*n* = 220/3292, 6.49%, CI: 5.71, 7.36%), after completion of FY1 (*n* = 1101/3292 32.46%, CI: 30.90, 34.05%) or after FY2 (*n* = 2071/3292, 61.06%, CI: 59.40, 62.68%). Figure 2a demonstrates the distribution of these intentions across UK medical schools, relative to total response rates per school. Notably, Ulster University had the highest proportion of students considering emigration (45.45%), in contrast to Edge Hill, where 19.64% held similar intentions. Among students intending to emigrate, 49.56% (*n* = 1681, CI: 47.88, 51.24%) planned a return to the UK after a few years abroad, while 7.87% (*n* = 267, CI: 7.01, 8.83%) expected to return after completing their medical training abroad. The remaining 42.57% (*n* = 1444, CI: 40.92, 44.24%) expressed no plans to return to practise in the UK, as demonstrated in Fig. 2b.

**Fig. 3:**
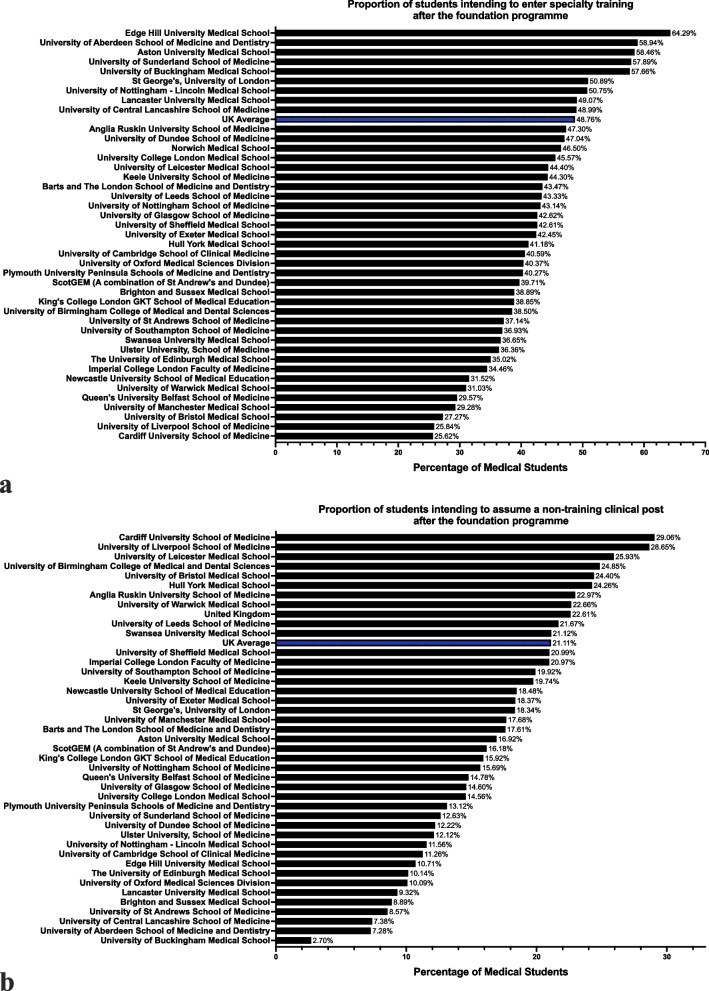
Distribution of Post-Foundation Programme Career Intentions Among UK Medical Students by School. **a** illustrates the proportion of students at each UK medical school intending to enter specialty training immediately following the Foundation Programme. **b** presents the proportion of students planning to enter non-training clinical roles (comprising ‘F3’ year roles, junior clinical fellowships, clinical teaching fellowships, or locum positions) in the UK after FY2

Of the 8806 respondents intending to complete both FY1 and FY2, 48.76% (*n* = 4294, CI: 47.72, 49.81%) planned to enter specialty training in the UK immediately thereafter; 21.11% (*n* = 1859, CI: 20.27, 21.98%) intended to enter a non-training clinical job in the UK (commonly comprising an ‘F3’ year, including a junior clinical fellowship or clinical teaching fellowship, or in locum roles). These ‘non-training’ roles, although valuable for gaining clinical experience, are largely standalone posts which do not contribute to accreditation within medical specialties. The school with the highest proportion of responses indicating plans to enter specialty training immediately after FY2 was Edge Hill (64.29%), whereas at Cardiff only 25.62% shared this intention. Cardiff students were also most likely to plan to enter non-training clinical posts after FY2, at 29.06%. Students from the University of Buckingham were, by far, the least likely to look to pursue non-training posts (2.70%). Figure 3a and b present the distribution of these intentions across UK medical schools.

**Fig. 4:**
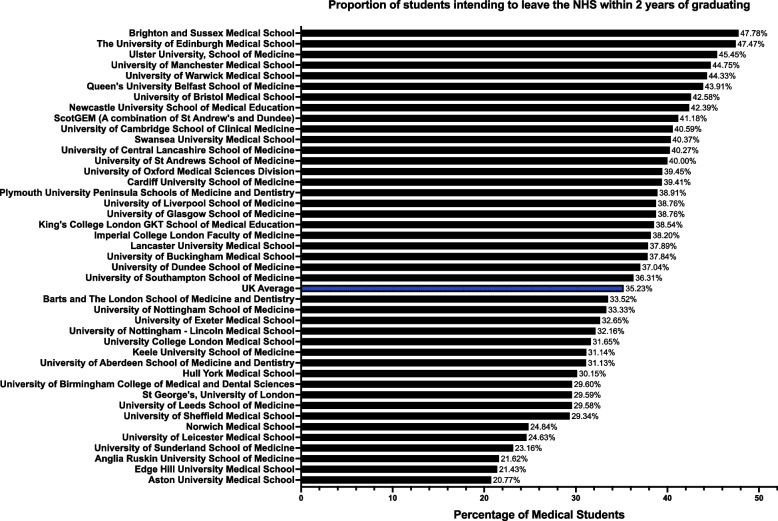
Proportion of UK Medical Students Intending to Leave the NHS Within 2 Years of Graduation, by School

In total, 35.23% (3695/10,486) of medical students intend to leave the NHS within 2 years of graduating, either to practise abroad or leave medicine. Respondents from Brighton and Sussex Medical School expressed this intention most often (47.78%), whilst those from Aston Medical School were the least likely to do so (20.77%) (Fig. 4).

**Fig. 5:**
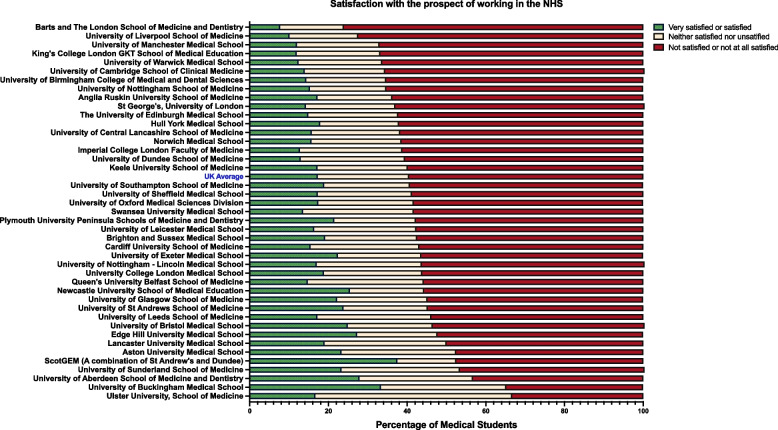
Medical Students’ Overall Satisfaction with the Prospect of Working in the NHS, by School. The figure illustrates the variation in levels of career satisfaction across UK medical schools

To better ascertain the medical student population’s sentiments towards working in the NHS, respondents were asked to share their degree of satisfaction with several factors. Likert scale matrices were employed, with options ranging from ‘Very satisfied’ to ‘Not at all satisfied’. An important aspect was students’ overall satisfaction with the prospect of working within the NHS, with which only 17.26% of students were either satisfied or very satisfied. This figure varied substantially by institution as illustrated in Fig. 5. Surveyed students from Barts and the London, Liverpool, and King’s College London GKT schools of medicine were the most dissatisfied, with dissatisfaction rates of 76.07, 72.48 and 66.84% respectively. Conversely, students from Aberdeen (43.27%), Buckingham (34.78%) and Ulster medical schools (33.33%) were those least dissatisfied with the prospect of working in the NHS.

## Discussion

### Principal findings

This study identified considerable institutional variation in students’ career intentions and sentiment about their future careers.

Our results show that, in each UK medical school, over a fifth of participating medical students intend to leave the NHS within 2 years of graduation – and in some medical schools, this figure was approximately half. Nationally, this figure surpassed a third of surveyed medical students. Most would-be leavers plan to emigrate, many permanently, while a notable minority of respondents plan to leave the profession altogether. Here, we consider possible reasons for these trends, and offer potential means of adapting medical schools to avert the loss of these medics from the NHS workforce.

The levels of satisfaction among medical students concerning their prospective employment within the NHS displayed marked disparities, influenced potentially by institutional factors. In certain schools, up to 76% of students expressed dissatisfaction with the prospect of a career within the NHS, contrasted with the 48% recorded in others. The national average of 60% dissatisfaction is concerning and warrants further investigation to identify the underlying causes of this marked variability across different medical schools. Understanding the specific factors influencing medical students’ satisfaction levels could be critical in developing strategies to improve their perceptions of careers in the NHS.

### Differing career sentiment between medical schools

Many differences exist between medical schools, some inherent or incidental, and others the result of decisions taken by medical faculties. Naturally, there is variation by geography, in the clinical environments and patient populations to which students are exposed, or in differences in the NHS between the UK’s devolved nations. The composition of the student body, in terms of various demographic characteristics also differs considerably between schools (Supplemental Figs. 1a-d). Additionally, despite meeting minimum standards set by the GMC, medical schools are distinct in their curriculum delivery and priorities, culture, and other factors. This ‘hidden curriculum’ can be influential in students’ outlook towards medicine and their careers [9]. Medical schools’ autonomy extends to setting local recruitment practices, leading to differences in entry requirements and favoured attributes for which candidates are selected [10].

### Curriculum focus and its influence

Certain faculties may favour students for academic potential or other attributes that may not necessarily correspond to their aptitude or interest in clinical medicine. At these schools, medical curricula may be more science-focused, such as by employing the ‘traditional’ model of medical education which firmly separates preclinical and clinical studies. During the early years of study, in which clinical exposure is low, students may find themselves detached from the medical field and begin considering alternative careers. This may be especially true where intercalated degrees form mandatory components of the curriculum – the receipt of which would enable pursuit of graduate roles or postgraduate degrees. Moreover, some institutions emphasising academic achievement may offer academic opportunities which could further distance those enrolled from the profession. For instance, previous graduates of MB/PhD programmes, an option to intercalate a PhD degree offered by only a limited number of universities, have gone onto careers in academia, industry, and business [11, 12].

### Recruitment practices

Despite the inherent importance of academic ability, it is important to recognise that a ‘good’ doctor requires a balance of various attributes including empathy, resilience, and communication skills. Furthermore, a clear understanding and realistic expectations of the profession are critical. The possible discrepancy between academic aptitude and the day-to-day reality of medical practice may be a contributing factor to the observed trends of students contemplating leaving the profession. Therefore, ensuring a balanced and holistic approach in selection processes could contribute to cultivating a workforce committed to pursuing medical practice in the NHS long term. Currently, prospective students undergo varying forms of interviews, which, due to their brevity and the substantial volume of applications, may not adequately capture a candidate’s realistic expectations and motivations towards a medical career. To increase the robustness of the selection process, medical schools should consider revisiting the structure of their interview processes, potentially incorporating methods to more accurately assess applicants’ understanding and enthusiasm for a medical career within the NHS more accurately. This approach could include comprehensive discussions focusing on the complexities and realities associated with a medical career [13]. Moreover, there are relevant differences in institutions’ selection criteria, with some valuing extracurricular activities, while some place greater emphasis on personal statements more, and others prioritise results achieved in admission exams [10]. Implementing such changes in the recruitment process can be a proactive step towards retaining talent within the NHS and encouraging more students to envisage a fulfilling career within the medical profession.

### Institutional reputation

Respondents from institutions which place highly in national and international university rankings exhibited a greater propensity to consider leaving the profession [14, 15]. Notably, the universities of Cambridge (8.59%), Oxford (8.26%), and Imperial College London (8.24%) led this trend. Attending these, and other, historically prestigious schools, may boost non-clinical career opportunities, so their students may be attracted to the perceived benefits of alternative careers over those in clinical practice. This institutional reputation may have initially attracted some students, for whom the career opportunities outside clinical practice now offer more compelling options compared to working in the NHS. This, coupled with growing reports of doctors looking to leave the health service, may partly explain the trend observed [3]. However, it is important to note that this phenomenon is neither new nor limited to the UK, with a 2001 study identifying growing numbers of medical students in the United States intending to pursue non-clinical, non-academic careers over time [16]. Notably, only four schools had 0% of students intending to leave the profession.

### Demographic influences

Moreover, the composition of the student body, particularly in terms of demographic makeup may represent another potential influence on career intentions. For instance, if data indicate that students from certain demographics were more likely to pursue a certain career path, a school with a higher proportion of such students may appear to exhibit a similar inclination. It is important to note that these tendencies may be reflective of broader societal and demographic differences, rather than factors intrinsic to the respective institutions. A deeper analysis of demographic nuances may elucidate the intricate interplay of background and career choices, offering valuable insights for future policy and institutional strategies. Furthermore, it would be prudent to recognise that certain students, particularly those from widening participation backgrounds, may have limited agency regarding the career pathway they pursue. For some, this limitation may be financial in nature or due to caring responsibilities, while for others it may be more strongly related to the awarding gap [17].

### Proposed solutions and future directions

Our findings underscore the need to explore the reasons for the observed disparities in students’ career sentiment across medical schools. Using this information, medical courses may be adapted to improve students’ feelings about their future medical careers in the NHS or otherwise. As students’ perspectives are guided by their educational experiences, undergraduate training they deem suboptimal could contribute to a diminished enthusiasm for a career in medicine. Higher standards of teaching may increase interest and engagement in the medical profession, while inadequate teaching quality could engender frustration and disillusionment. Unsatisfied students may opt to pursue alternative careers or relocate to destinations where they perceive education and training standards to be higher [18]. To substantiate this, further studies could endeavour to quantify perceptions towards teaching standards at medical school and the impact of teaching quality on students’ career choices, potentially guiding improvements in curriculum design and faculty development.

It is important to note that many respondents will have been studying medicine during the COVID-19 pandemic. During this period, medical schools had the difficult task of balancing infection risk with maintaining educational standards. Centres will have differed in their approach, and negative experiences - educational or otherwise - from this period may have adversely influenced students’ attitudes towards medicine [19].

Furthermore, the structure or variety of clinical placements used by some medical schools could more effectively convey a positive outlook of medical careers or the NHS. This is often contingent on the clinical environments in which medical students rotate. For instance, limited exposure to certain specialties or sub-specialties—only available at select centres—may inadvertently obscure potentially rewarding career paths. Similarly, limited opportunities in rural medicine, public health, or other non-hospital-based pathways may also achieve the same effect [20]. Spaces and learning opportunities may also be shared with increasing cohort sizes or, depending upon geography, with students from other medical schools, potentially diluting learning opportunities [21]. Staffing levels, workplace culture and health outcomes also vary geographically, both within and between the UK’s devolved nations [22–25]. These factors inform students’ perceptions of the career and may contribute to their decision-making. To mitigate this, medical faculties would benefit from establishing or expanding student feedback mechanisms. The objective is to identify factors affecting training experiences and to ensure equitable access across the UK, irrespective of the medical school attended.. Such engagement may also reveal which career paths are under-explored in individual medical curricula. In response to students’ views, or from faculties’ own understanding of where these deficits may lie, schools may consider offering means of addressing this, such as through optional specialty taster days.

Where higher proportions of students expressed interests in either relocating to work abroad or in leaving the profession entirely, there may be benefit in fostering a culture of mentorship and guidance around medical careers. Mentorship can support students to navigate systems used during applications for increasingly competitive specialty training programmes [26, 27]. Guidance from medics acquainted with these processes can support students to pursue their preferred specialty and could consequently reduce attrition by improving their perceived career prospects.

### Findings in context

The AIMS study highlighted a wide range of factors which contribute to medical students’ career sentiment and their intended career trajectory [5]. Here, we explored the role of medical schools in this complex equation and, although influential, this must be considered in that wider context. While national policy reform addressing factors such as remuneration and working conditions are required to reverse current trends in students’ career intentions, the strategies proposed in this manuscript may serve to address regional disparities.

### Limitations

Despite the AIMS study constituting the largest ever study of UK medical students, due to the methods of dissemination, the number of students who saw the invitation to participate in the study is unknown, and therefore we are unable to calculate the response rate. Consequently, the sample may have been subject to selection bias, possibly driven by greater response rates among students with existing interests in this subject. Additionally, the questions in our survey instruct students to be definitive even when they might not yet have formulated their career plans, a not-improbable situation, particularly for those in the early years of medical school.

Moreover, being a cross-sectional study, it is not possible to comment on changes to medical students’ career sentiment with time. Although informed by their undergraduate training and experiences therefrom, at the time of participation, respondents had not yet worked as medical doctors. As such, their opinions may change once immersed in the career and working in the health service. In anticipation of this limitation, the questionnaire sought consent for a planned follow-up study, to which a 71.29% positive response rate was captured. It is hoped that this study’s findings may be validated by tracking changes in sentiment over time.

Importantly, there was also variability in the number of responses achieved from each medical school. This occurred despite recruitment of a large medical student collaborator network. This discrepancy might be attributed to various factors, including the approach of dissemination undertaken by university or medical school administrators, the design of clinical placements, or the presence and influence of local student societies, among other considerations. To avoid potential misrepresentation due to inadequate sample sizes, we opted to exclude data from the two medical schools that obtained fewer than 30 responses.

## Conclusion

While the broader trends of medical students intending to leave the NHS within 2 years of graduating are concerning, the variation in career sentiment across UK medical schools requires consideration. This analysis implicates a complex interplay of factors—ranging from curriculum focus and cohort demographics to recruitment strategies, teaching quality, and clinical experience—in shaping these career intentions. Such variation in career sentiment between institutions may be indicative of deeper issues, possibly rooted in educational approaches and experiences at undergraduate level - on which the potential impact of the COVID-19 pandemic should be noted.

It is evident that approaches taken to recruitment, educational framework, and support within medical schools require reassessment. Subsequent investigations should examine the underlying causes of disparities in career sentiment by institution, aiming to cultivate resilience, dedication, and - critically - professional fulfilment among the future medical workforce in the UK.

### Supplementary Information

**Supplementary Material 1.**

**Supplementary Material 2.**

**Supplementary Material 3.**

**Supplementary Material 4.**

**Supplementary Material 5.**

## Data Availability

The datasets used and/or analysed during the current study are available from the corresponding author on reasonable request once all planned subsequent analyses are completed.

## References

[1] Ibrahim H, Nair SC, Shaban S, El-Zubeir M. Reducing the physician workforce crisis: career choice and graduate medical education reform in an emerging Arab country. Educ Health (Abingdon). 2016;29(2):82–8. 10.4103/1357-6283.188716. PMID: 27549644. 27549644 10.4103/1357-6283.188716

[2] General Medical Council. The state of medical education and practice in the UK. The workforce report; 2022.

[3] Waters A. A third of junior doctors plan to leave NHS to work abroad in next 12 months. BMJ. 2022;379:3066. 10.1136/bmj.o3066 36581347

[4] BMA. Catastrophic crisis facing NHS as nearly half of hospital consultants plan to leave in next year, WARNS. 2022. Available: https://www.bma.org.uk/bma-media-centre/bma-report-reveals-potentially-catastrophic-crisis-in-hospital-consultant-workforce-levels. Accessed 17 Apr 2024.

[5] Ferreira T, Collins AM. Feng O the AIMS collaborative, et al career intentions of medical students in the UK: a national, cross-sectional study (AIMS study). BMJ Open. 2023;13:e075598. 10.1136/bmjopen-2023-075598. 10.1136/bmjopen-2023-075598 PMC10496670 37699638

[6] Rourke J. How can medical schools contribute to the education, recruitment and retention of rural physicians in their region? Bull World Health Organ. 2010;88(5):395–6. 10.2471/BLT.09.073072. 20461207 10.2471/BLT.09.073072 PMC2865664

[7] Ferreira T, Collins AM, Horvath R. Ascertaining the career intentions of medical students (AIMS) in the United Kingdom post graduation: protocol for a mixed methods study. JMIR Res Protoc. 2023;12:e45992. 10.2196/45992. 37335615 10.2196/45992 PMC10337401

[8] von EE, Altman DG, Egger M, et al. The strengthening the reporting of observational studies in epidemiology (STROBE) statement: guidelines for reporting observational studies. BMJ. 2007;335:806–8. 17947786 10.1136/bmj.39335.541782.AD PMC2034723

[9] Lempp H, Seale C. The hidden curriculum in undergraduate medical education: qualitative study of medical students’ perceptions of teaching. BMJ. 2004;329:770. 10.1136/bmj.329.7469.770. 15459051 10.1136/bmj.329.7469.770 PMC520997

[10] Medical Schools Council Medical school entry requirements for 2024 start [internet]. Medical Schools Council; [cited 2023 Sep 18]. Available from: https://www.medschools.ac.uk/studying-medicine/making-an-application/entry-requirements-for-2024-start#:~:text=On%20entry%2C%20applicants%20must%20havehigher%20than%20for%20those%20without.

[11] Cox TM, Brimicombe J, Wood DF, Peters DK. The Cambridge bachelor of medicine (MB)/doctor of philosophy (PhD): graduate outcomes of the first MB/PhD programme in the UK. Clin Med (Lond). 2012;12(6):530–4. 10.7861/clinmedicine.12-6-530. PMID: 23342406; PMCID: PMC5922592. 23342406 10.7861/clinmedicine.12-6-530 PMC5922592

[12] Barnett-Vanes A, Ho G, Cox TM. Clinician-scientist MB/PhD training in the UK: a nationwide survey of medical school policy. BMJ Open. 2015;5:e009852. 10.1136/bmjopen-2015-009852. 10.1136/bmjopen-2015-009852 PMC4710833 26719322

[13] Ferreira T. Beyond government accountability: the role of medical schools in addressing the NHS workforce crisis. J R Soc Med. 2023;116(11):395–8. 37905493 10.1177/01410768231209021 PMC10686201

[14] QS World University Rankings 2023. Top global universities [Internet]. Top Universities; 2023. [cited 2023 Sep 18]. Available from: https://www.topuniversities.com/university-rankings/world-university-rankings/2023?&tab=indicators .

[15] Times Higher Education. World University Rankings 2023 [Internet]. 2023. [cited 2023 Sep 18]. Available from: https://www.timeshighereducation.com/world-university-rankings/2023/world-ranking .

[16] Richard GV, Nakamoto DM, Lockwood JH. Medical Career Choices: Traditional and New Possibilities. JAMA. 2001;285(17):2249–50. 10.1001/jama.285.17.2249-JMS0502-3-1. 11325331 10.1001/jama.285.17.2249-JMS0502-3-1

[17] Brown C, Goss C, Sam AH. Is the awarding gap at UK medical schools influenced by ethnicity and medical school attended? A retrospective cohort study. BMJ Open. 2023;13(12):e075945. 10.1136/bmjopen-2023-075945 PMC10753756 38086586

[18] Gouda P, Kitt K, Evans DS, et al. Ireland’s medical brain drain: migration intentions of Irish medical students. Hum Resour Health. 2015;13:11. 10.1186/s12960-015-0003-9. 25889783 10.1186/s12960-015-0003-9 PMC4363465

[19] Wilcha R. Effectiveness of virtual medical teaching during the COVID-19 crisis: systematic review. JMIR Med Educ. 2020;6(2):e20963 https://mededu.jmir.org/2020/2/e20963. 10.2196/20963 . 33106227 10.2196/20963 PMC7682786

[20] Pathman DE, Konrad TR, Ricketts TC 3rd. Medical education and the retention of rural physicians. Health Serv Res. 1994;29(1):39–58 PMID: 8163379; PMCID: PMC1069987. 8163379 PMC1069987

[21] Roberts N, Bolton P. Medical school places in England from September 2018. London: House of Commons Library; 2017.

[22] NHS digital health. NHS workforce statistics. 2023. https://digital.nhs.uk/data-and-information/publications/statistical/nhs-workforce-statistics/february-2023. Accessed 17 Apr 2024.

[23] Dixon-Woods M, Baker R, Charles K, Dawson J, Jerzembek G, Martin G, McCarthy I, McKee L, Minion J, Ozieranski P, Willars J. Culture and behaviour in the English National Health Service: overview of lessons from a large multimethod study. BMJ Qual Saf. 2014;23(2):106–15. 10.1136/bmjqs-2013-001947 PMC3913222 24019507

[24] Office for National Statistics. "Health in England: 2015 to 2020." 2022. Available from: https://www.ons.gov.uk/peoplepopulationandcommunity/healthandsocialcare/healthandwellbeing/bulletins/healthinengland/2015to2021. Accessed 17 Apr 2024.

[25] Report by the Comptroller and Auditor General. Healthcare across the UK: a comparison of the NHS in England, Scotland, Wales and Northern Ireland. London: National Audit Office; 2012. https://www.nao.org.uk/wp-content/uploads/2012/06/1213192es.pdf. Accessed 17 Apr 2024.

[26] Best J. The growing bottlenecks in specialty training. BMJ. 2023;382:1732. 10.1136/bmj.p1732. 37536730 10.1136/bmj.p1732

[27] Ferreira T. Escalating competition in NHS: implications for healthcare quality and workforce sustainability. Postgrad Med J. 2024:qgad131. 10.1093/postmj/qgad131 38204332

