# Correction: The role of medical schools in UK students’ career intentions: findings from the AIMS study

**DOI:** 10.1186/s12909-024-05800-9

**Published:** 2024-07-24

**Authors:** Tomas Ferreira, Alexander M. Collins, Arthur Handscomb, Dania Al-Hashimi, the AIMS Collaborative

**Affiliations:** 1 School of Clinical Medicine, University of Cambridge, Cambridge, UK; 2 School of Public Health, Faculty of Medicine, Imperial College London, London, UK; 3 Bristol Medical School, University of Bristol, Bristol, UK; 4 Shefeld Medical School, University of Shefeld, Shefeld, UK

**Correction: BMC Med Educ (2024) 24, 604**

**https://doi.org/10.1186/s12909-024-05366-6**

Following publication of the original article [1], the authors identified an error in the affiliation of the AIMS collaborator Vaishvi Dalal.

The incorrect affiliation is: School of Medicine, University of Glasgow, Glasgow

The correct affiliation is: UCL Medical School, University College London, London.
