# Supplementary material for: The role of medical schools in UK students’ career intentions: findings from the AIMS study
Source: BMC Med Educ. 2024 May 31;24:604. doi: 10.1186/s12909-024-05366-6 (PMC11143605; doi:10.1186/s12909-024-05366-6)
Supplement: Supplementary file 1 — Supplementary Material 1. [file 12909_2024_5366_MOESM1_ESM.pdf]

Supplemental Figures 1a-d: Demographic Composition of Students Across UK Medical Schools. Figure 1a illustrates the breakdown of students at each medical school by ethnic identity. Figure 1b delineates the gender distribution across each medical school. Figure 1c presents the educational background of students at each school. Figure 1d demonstrates respondents' previous schooling by medical institution.
