# Supplementary figures and images for: The role of medical schools in UK students’ career intentions: findings from the AIMS study
Source: BMC Med Educ. 2024 May 31;24:604. doi: 10.1186/s12909-024-05366-6 (PMC11143605; doi:10.1186/s12909-024-05366-6)

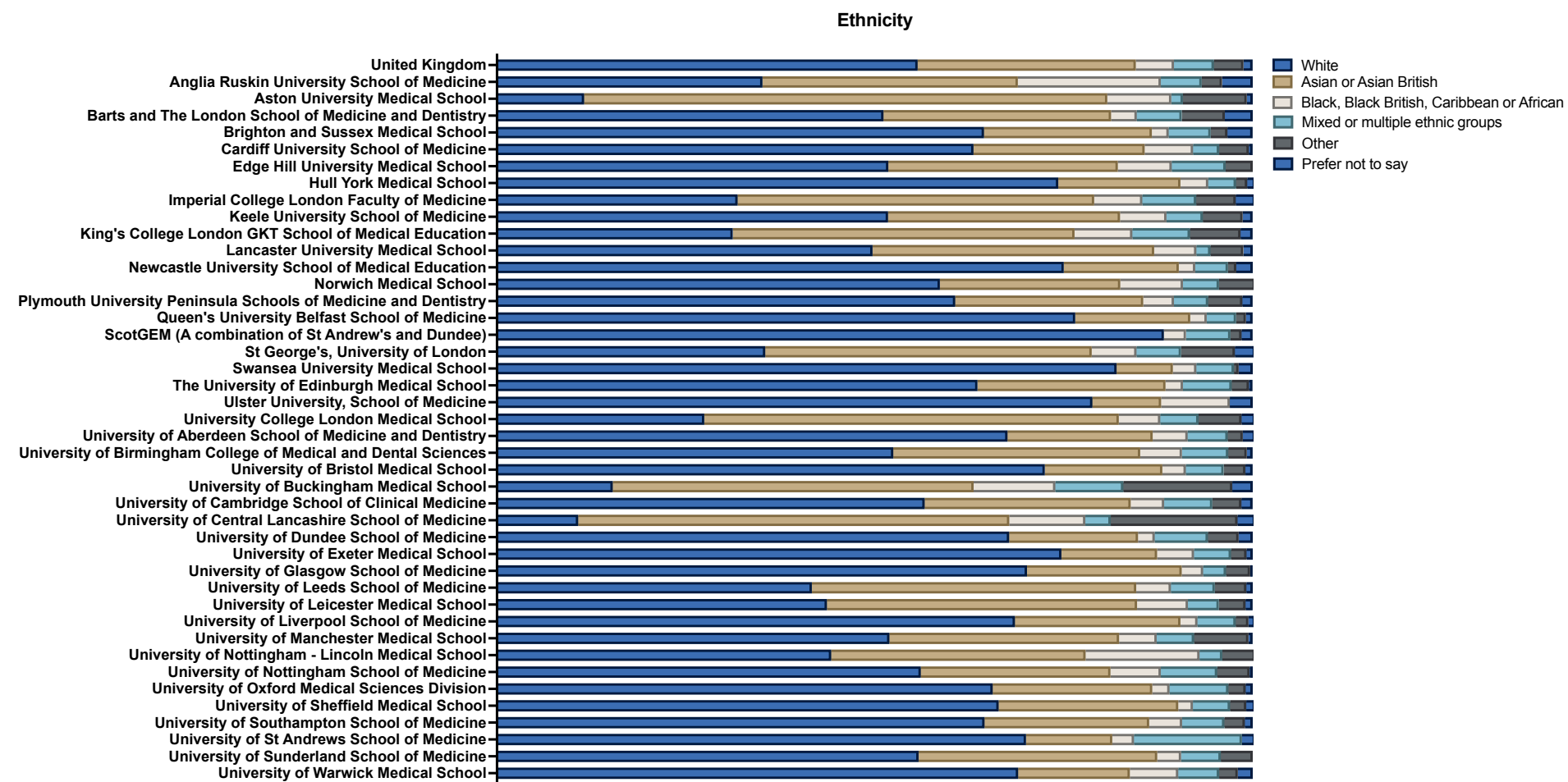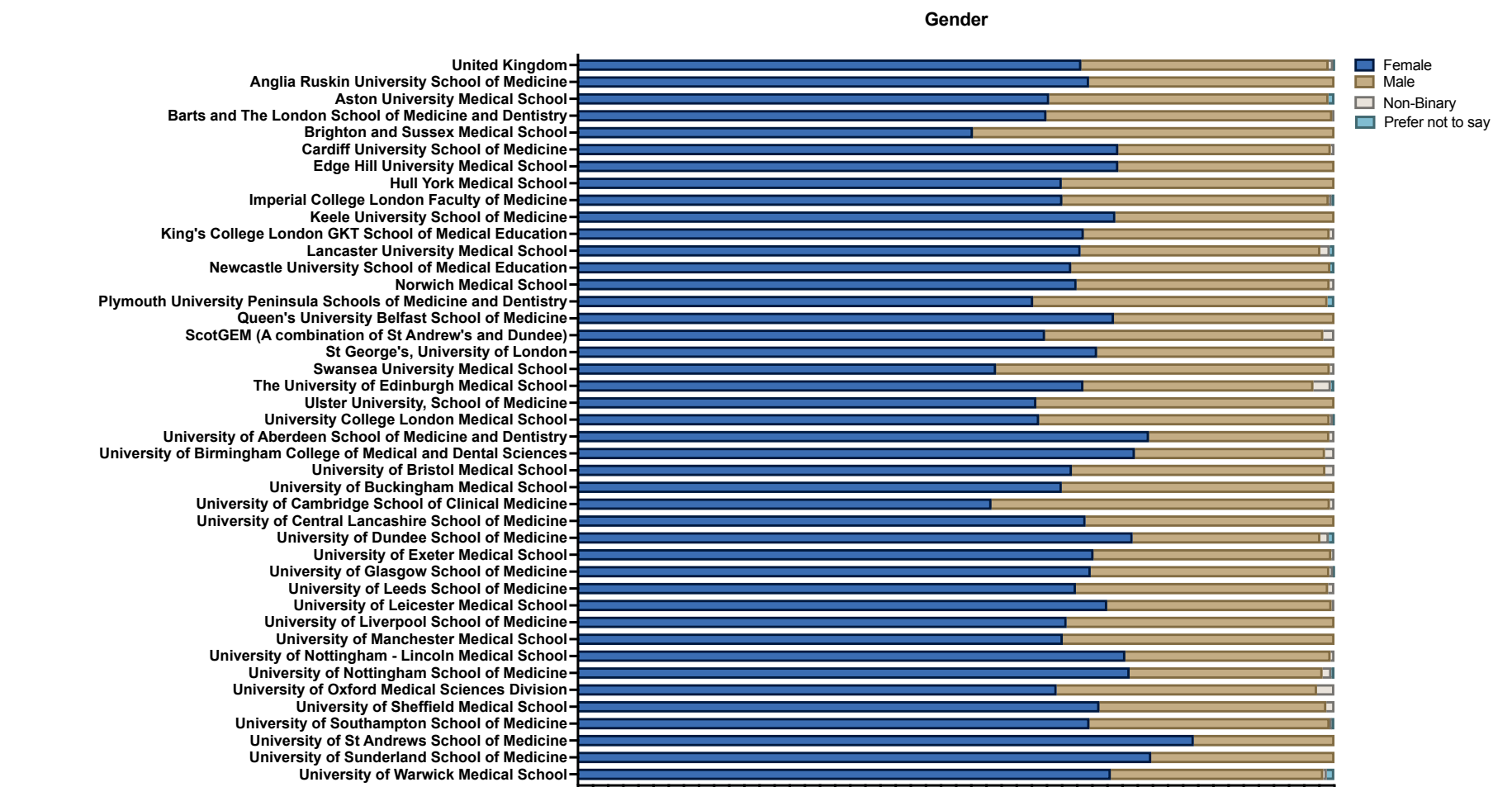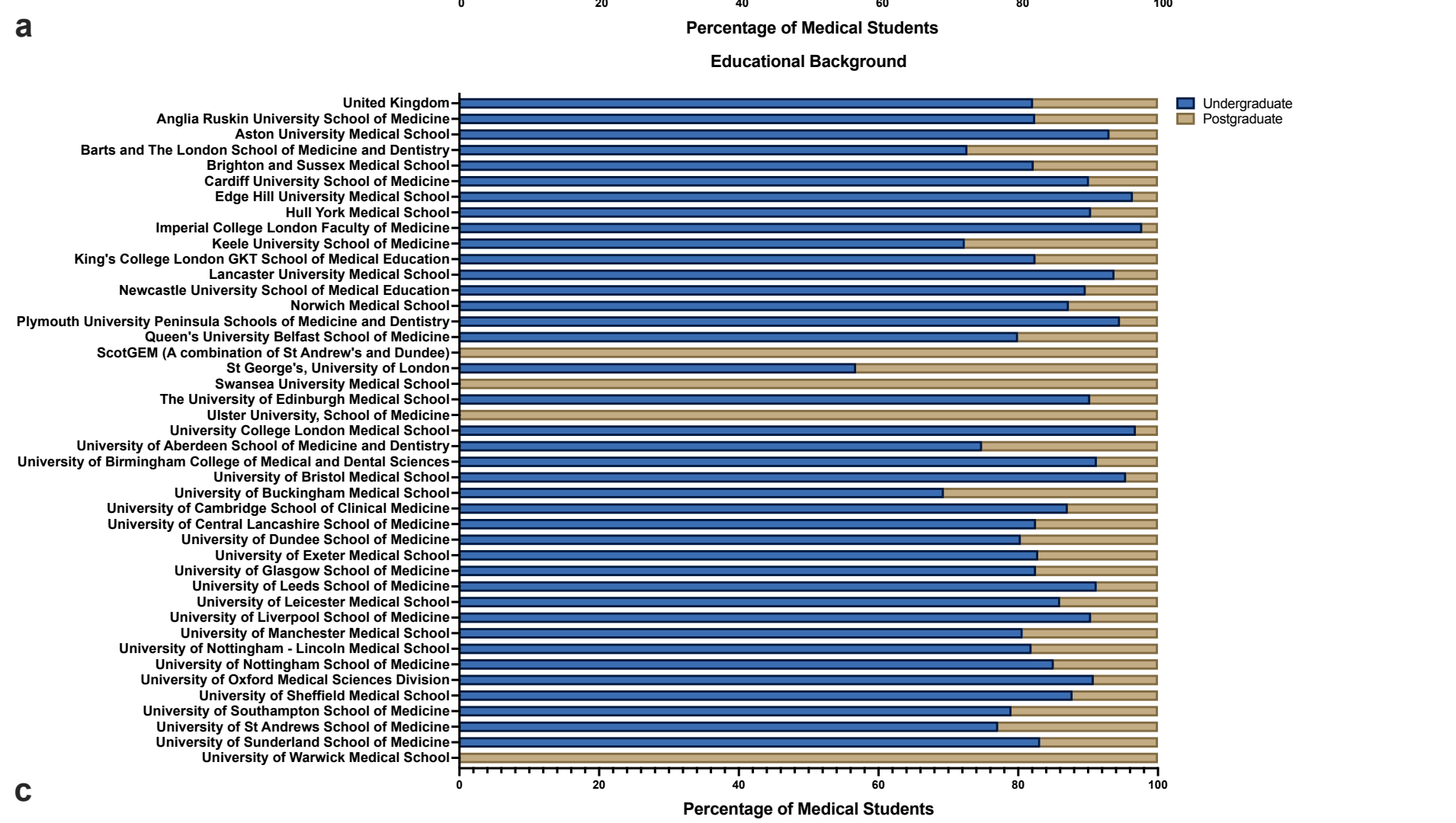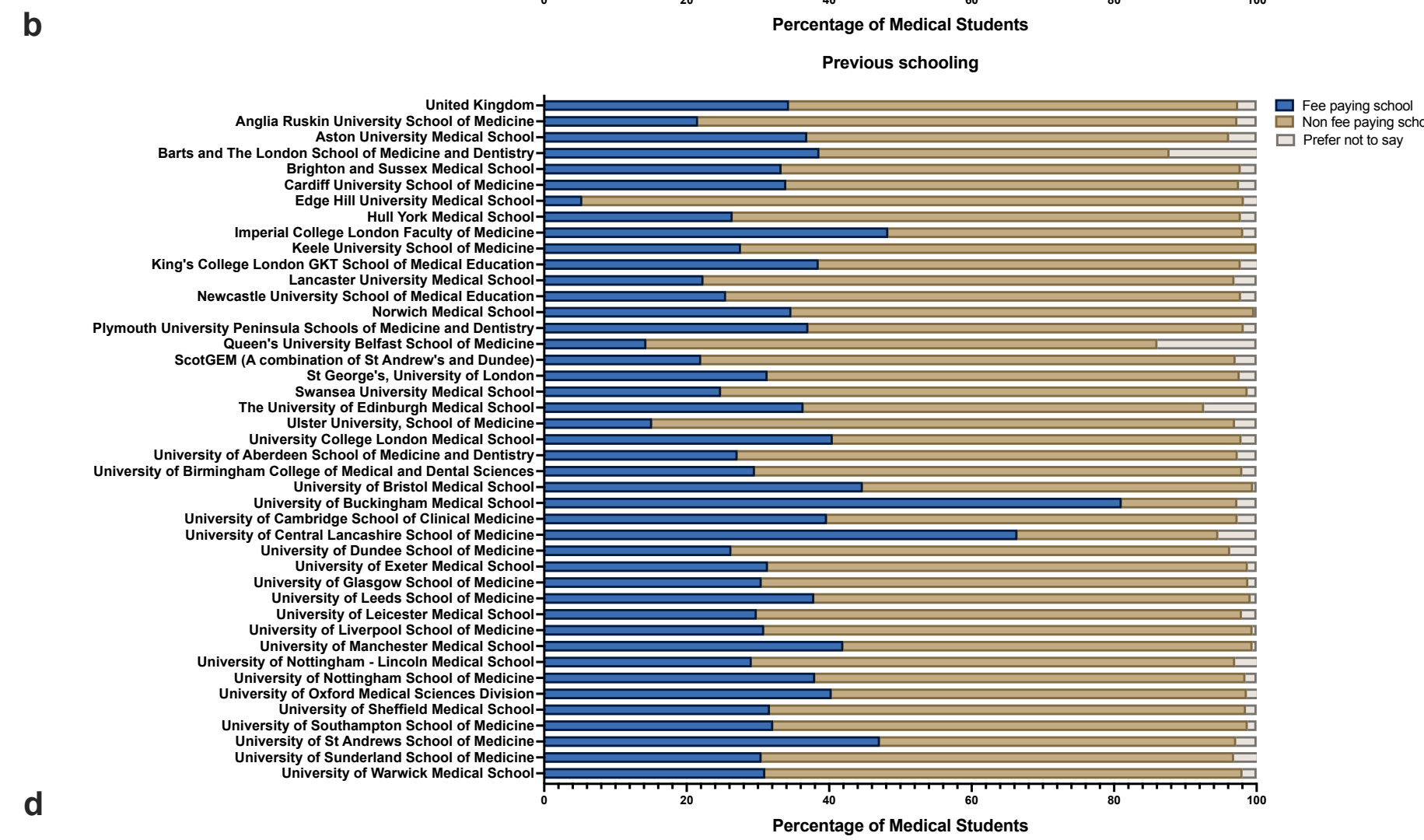

Supplement: Supplementary file 2 — Supplementary Material 2. [file 12909_2024_5366_MOESM2_ESM.pdf]
